# Sex Differences in Emotional Evaluation of Film Clips: Interaction with Five High Arousal Emotional Categories

**DOI:** 10.1371/journal.pone.0145562

**Published:** 2015-12-30

**Authors:** Antonio Maffei, Valentina Vencato, Alessandro Angrilli

**Affiliations:** 1 Department of General Psychology, University of Padova, Padova, Italy; 2 IN CNR Institute of Neuroscience, Padova Section, Padova, Italy; University of Bologna, ITALY

## Abstract

The present study aimed to investigate gender differences in the emotional evaluation of 18 film clips divided into six categories: Erotic, Scenery, Neutral, Sadness, Compassion, and Fear. 41 female and 40 male students rated all clips for valence-pleasantness, arousal, level of elicited distress, anxiety, jittery feelings, excitation, and embarrassment. Analysis of positive films revealed higher levels of arousal, pleasantness, and excitation to the Scenery clips in both genders, but lower pleasantness and greater embarrassment in women compared to men to Erotic clips. Concerning unpleasant stimuli, unlike men, women reported more unpleasantness to the Compassion, Sadness, and Fear compared to the Neutral clips and rated them also as more arousing than did men. They further differentiated the films by perceiving greater arousal to Fear than to Compassion clips. Women rated the Sadness and Fear clips with greater Distress and Jittery feelings than men did. Correlation analysis between arousal and the other emotional scales revealed that, although men looked less aroused than women to all unpleasant clips, they also showed a larger variance in their emotional responses as indicated by the high number of correlations and their relatively greater extent, an outcome pointing to a masked larger sensitivity of part of male sample to emotional clips. We propose a new perspective in which gender difference in emotional responses can be better evidenced by means of film clips selected and clustered in more homogeneous categories, controlled for arousal levels, as well as evaluated through a number of emotion focused adjectives.

## Introduction

Sexual dimorphism and gender behavioral differences are widespread in nature [1]: an evolutionistic analysis may explain sexual differences as due to inherited traits linked to social-adaptive roles [2]. Thanks to the growth in brain imaging techniques in the last few decades, it has now been acknowledged that men’s and women’s brains are structurally, chemically, and functionally different, and this knowledge allows for an understanding of gender differences in both the cognitive and emotional domains [3]. Distinct morpho-functional features in the limbic and paralimbic brain structures of women’s brains may help to explain their greater vulnerability to emotional stress and higher risk of developing a mood disorder [4,5]. In line with this clinical risk, in past literature, a first hypothesis on gender emotional difference tried to demonstrate that women report emotional experiences with greater intensity than men [6,7]. The following research demonstrated that women, compared to males, are more sensitive to stress and reported greater self-perceived unpleasantness, as well as greater central and peripheral physiological activation to aversive stimuli [8–10]. Conversely, men showed an increased response especially to erotic appetitive stimuli [11,12]. It is worth noting that the majority of previous studies were based on the use of emotional stimuli within the broader classification of pleasant and unpleasant categories, while studies based on film clips tried to distinguish among more specific categories of emotional material [13–16]. Indeed, one of the reasons for using film clips as stimuli in research is that some complex emotions, such as sadness, anger, or compassion, do not fully develop in the few seconds of presentation typically used for slides. In addition, film clip presentation has been proved to elicit a stronger affective state in the viewer and, at the same time, to be one of the most ecological tools available for the manipulation of emotions in the experimental setting, as it resembles real-world emotional exposure [17,18].

Within the cluster of aversive stimuli, a greater reactivity to fearful and disgusting elicitors was found among women compared to men [19,20], while, for sad stimuli, gender difference was found at the physiological cortical level but not in self-ratings [21]. Concerning the influence of gender on positive emotional states, erotic stimuli represents a specific biologically relevant category, since scenes depicting nudity and sexual activity are able to trigger greater emotional responses in men compared to women, with males’ subjective evaluation indicating greater arousal and pleasantness [22]. In addition, participant’s gender and sexual orientation evidenced clear and specific emotional responses largely varying depending on the erotic stimuli composition (opposite- vs same- sex couples, males vs females erotic portraits) [23]. These subjective results were accompanied by greater sympathetic activation to erotic stimuli in males with respect to females [11]. Past studies based on film clips and specific emotional categories had some limitations, including the lack of control for the arousal-activation level: differences found among different categories (e.g. Erotic, Sadness, or Fear) might be due to underlying large arousal differences, which means that direct comparison among several emotions is complicated by this additional important variable and its possible interaction with gender. A second issue concerns film duration which substantially varied across emotional categories while psychometrically makes sense to equate/control stimulus duration as a further possible confound-uncontrolled variable

In the present research, we aimed to increase the current knowledge of gender difference in emotion by extending the research to some specific emotional categories that, according to our hypothesis, are biologically relevant and can be substantially influenced by gender. In the new adopted paradigm, some new categories of emotional stimuli were added to the ones previously studied, namely Erotic and Fear. Although sadness has been among the most investigated emotions in terms of film clips (see for review [14,24]), the present experiment split this category into two distinct (although partially overlapping) categories. Specifically, we were interested in investigating how men and women differ in their self-rating of emotional experience when exposed to film excerpts in which characters cry after loss or separation (Compassion category; we do not claim that this label encompasses all complex feelings associated with the many psychological, religious and philosophical meanings of the word compassion), and how these responses differ from those elicited by sad clips, in which characters do not cry (Sadness category). Since tears are a powerful social sign [25] that conveys information about one’s experience of physical and/or mental distress, seeing someone crying should trigger an automatic emotional response related to empathetic processes, which we expected to be different from that elicited by sadness without crying. More importantly, we expected to find a greater emotional response to Compassion in women, both in terms of experienced arousal and unpleasantness [26].

We also created a subtle distinction between different categories of clips depicting landscapes (either urban or natural), which, in the past, have been used as neutral clips. Indeed, while clips portraying urban environments were expected to represent true emotionally neutral stimuli, spectacular natural landscapes, especially when presented dynamically as in film clips, are able to elicit in the viewer a positive and contemplative state [27,28]. Therefore, in addition to the Erotic and Fear stimuli [11,29–31], which are biologically relevant and have rarely been compared in a study before, the present investigation measured the interaction between gender differences and four other emotional categories: Scenery, Compassion, Sadness, and Neutral. Greater but differentiated responses were hypothesized in women to the Fear, Sadness, and Compassion film clips, while larger responses were hypothesized in men to the erotic ones. For each category, self-rated emotional valence and arousal were measured together with other more specific dimensions elicited by the movies: distress, anxiety, excitement, embarrassment, and agitation.

## Materials and Methods

### Participants

Eighty-one healthy psychology students, 41 females (mean age ± S.D.: 21.88 ± 3.5 years) and 40 males (mean age ± S.D.: 22.46 ± 2.30 years), were recruited from an introductory psychology course to participate in the present study and were rewarded with course credit. All the participants were Italians had normal or corrected-to-normal vision and were screened to exclude any neurological or psychiatric conditions. The study was approved by the Psychology Ethics Committee, University of Padova. The investigation has been conducted according to the principles expressed in the Declaration of Helsinki.

### Stimuli

Film clips were selected from a previously validated larger sample of emotional stimuli, which originally included 39 film clips rated on several emotional dimensions by about 200 participants (the results of the validation research will be reported in another study). Eighteen excerpts belonging to six categories were selected for the present study: Erotic, Scenery, Neutral, Sadness, Compassion, and Fear. With the exclusion of the Neutral one, all clips were selected to induce a similar level of arousal. The reason for this was to minimize arousal differences among emotional conditions and to allow for a better comparison of the five emotional categories by controlling as much as possible arousal level. Each emotional category included three short excerpts selected from commercially available movies, making a total of eighteen clips. The length of the clips was approximately two minutes each (± 10 sec). With respect to past film validations, the selected clips were all extracted in High Resolution (HD) mode (1280x720 pixels). Details on the selected interval and its duration for each film are reported in the S1 File.

The Erotic category included three excerpts selected from *Monster’s Ball* (2001), *The Notebook* (2004), and *Lust*, *Caution* (2007), which portrayed heterosexual couples engaging in a sexual act. The Scenery category included clips depicting stunning views of natural landscapes, selected from the BBC’s documentary series *Planet Earth* (2006), while the Neutral category included excerpts drawn from urban documentaries about London, Paris, and New York City. The Sadness category comprised clips selected from *The Road* (2009), *K-19* (2002), and *Blood Diamond* (2006), which featured themes of desperation and helplessness. The Compassion category was characterized instead by scenes portraying loss and grief and a main character in tears. This category included clips selected from *The Pursuit of Happiness* (2006), *Armageddon* (1998), and the TV series *Lost* (2004). Finally, the Fear category comprised excerpts selected from *The Silence of the Lambs* (1991), *The Sixth Sense* (1999), and *Gothika* (2003). For all the clips but three the original audio was presented; in the three Scenery clips, to allow subjects to focus on the aesthetic content of the landscapes, the original speaker-based score typical of documentaries was replaced with ambient music.

The excerpts were edited using Adobe Premiere CS5 to achieve a standardized HD resolution (1280x720 pixels). The order of presentation of the excerpts was pseudo-randomized to avoid two excerpts belonging to the same category being consecutive. The stimuli were presented on a 23.6-inch 16:9 ratio full HD monitor and through stereo headphones.

### Procedure

Upon arrival, each participant was allowed to sit in a comfortable armchair and was given a general description of the experiment and the procedure in order to obtain the signed informed consent. Next, experimenters administered the emotional assessment to the participant, dimmed the lights, and the session started. After each clip, participants were asked, through on-screen instructions, to rate their emotional experience using analog scales, reported in the emotional assessment section. Fifty seconds were allowed for the completion of the scales after each film. In order to minimize any socially-related confounds, each participant took part to the experiment in a solitary session.

In the present experiment no data were collected on women’s phase of menstrual cycle and on sexual orientation of participants. In Italy 2.4% of population has a not-heterosexual orientation (ISTAT–Italian Statistic Institute, 2011), in line with UK statistics reporting 2.6% of population (ONS–Office for National Statistics, 2012). This probabilistically corresponds to about 2 out 80 non-heterosexual participants in our sample.

### Emotional Assessment

Participants were asked to rate first emotional valence (from maximum unpleasantness 1 to maximum pleasantness 9), then their self-perceived arousal (from minimum arousal/calmness 1 to maximum arousal 9) in response to each clip by means of the paper-and-pencil version of the Self-Assessment Manikin on a 1–9 scale [32]. Following the completion of the SAM, participants were asked to rate a series of emotional adjectives that described their feelings during the viewing of each clip. This was done using a 5-point scale from 1 (“Not at All”) to 5 (“Extremely”). The adjectives to be rated were presented in this order: Distressed, Excited, Embarrassed, Jittery, Anxious.

### Data Analysis

Separate mixed ANOVAs were computed for each dependent variable-scale included in the assessment. This resulted in the within-subjects variable, Emotional Category, featuring six levels (Erotic, Scenery, Neutral, Sadness, Compassion, and Fear), and the between-subjects variable Gender (Women vs Men). Newman-Keuls was used for post-hoc analyses and p was fixed at 0.05 and 0.01 levels of significance. All analyses were carried out using STATISTICA software (Statsoft). Concerning the emotional adjectives, a departure from normality was expected for these variables. For each emotional adjective, a rating above 1–2 was hypothesized only for one or two film categories, while for the other categories, the rating was not expected to vary from the minimum: this unavoidably leads to normality departure (and it was, indeed, found: Kolmogorov-Smirnov test was significant in several cells of each adjective). Since ANOVA allows for a comparison of all conditions, even in complex designs, and is also quite robust also when used with ordinal data that departs from normality [33,34], we decided to mainly use ANOVA analysis. In support of this use of ANOVA, additional analyses were carried out with non-parametric tests typically considered best suited for non-normal distributed data. Thus, the Wilcoxon for paired non-repeated comparisons and the Mann-Whitney U-test for repeated measures were carried out on the emotional adjective data. Additional analyses included the within-subjects correlation of self-reported arousal levels with the self-evaluated emotional adjective levels, and were carried out separately for the two groups. Raw data used for statistics are reported in Tables A to G in the S2 File.

## Results

### Emotional Valence

The ANOVA of emotional valence self-evaluation showed no effect of the Gender factor (F1,79 = 1.28, ns) and a main effect of Emotional Category (F5,395 = 72.01, p< 0.01). This indicated, in the post-hoc analysis, greater ratings (pleasantness) for Erotic and Scenery compared to Neutral film clips, and significantly lower valence ratings (unpleasantness) for Compassion and Sadness film clips compared to Neutral and Fear with respect to the other two unpleasant conditions (all p< 0.01). No difference was found in the comparisons between the Erotic and Scenery or between the Compassion and Sadness film clips.

**Fig 1:**
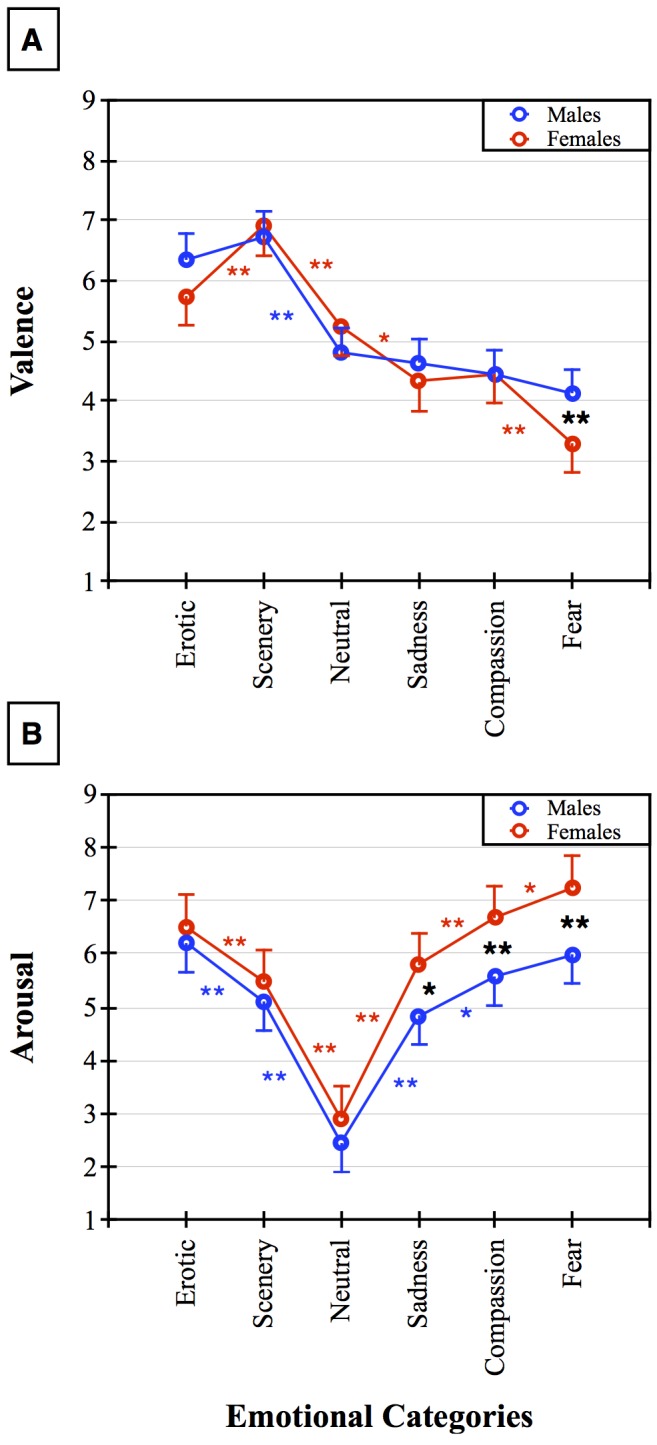
Panel A shows the ratings of emotional valence in response to the six emotional film categories measured on a 1–9 analogue scale in males and females. Asterisks indicate * p<0.05 and ** p< 0.01. Red indicates the within-females post-hoc comparisons. Blue: within-males post-hoc comparisons. Black: between-groups post-hoc comparisons. Panel B shows the ratings of self-perceived arousal in response to the six emotional film categories measured on a 1–9 analogue scale in males and females. Asterisks and colors are as above.

Also, the interaction between Gender and Emotional Category was significant (F5,395 = 2.77, p<0.05; see Fig 1A). In particular, women reported Fear clips as more unpleasant compared to men (Newman-Keuls post-hoc test, p<0.01). In addition, within group patterns were different for the two genders. Women rated Fear clips as significantly more unpleasant than Compassion, Sadness, and Neutral clips (Newman-Keuls post-hoc test for all three differences p<0.01). Furthermore, the Sadness and Compassion clips were rated as more unpleasant than the neutral ones (all p<0.05). The Scenery and Erotic clips were judged as more pleasant than the Neutral ones (p<0.05), but for women, the Erotic clips were less pleasant than the Scenery ones (p<0.01). A different pattern was found for males (Fig 1A) who did not show any difference among the Neutral clips and the three negative categories, Compassion, Sadness, and Fear. They rated the Erotic and Scenery clips as being more pleasant than all the other categories (p<0.01), but, unlike the women, they found Scenery and Erotic equally pleasant (Fig 1A).

### Arousal

The ANOVA of arousal showed a main effect of the Gender factor (F1,79 = 12.48, p<0.01), with women exhibiting an overall greater self-perceived arousal than men (Fig 1B). Also, the main effect of Emotional Category was significant (F5,395 = 120.76, p< 0.01), and greater arousal was found to both Erotic and Fear clips compared with all the other categories. Scenery, Compassion, and Sadness elicited significantly more arousal than the neutral clips (p<0.01), and Compassion induced more arousal than Sadness (p<0.01). The significant interaction between Gender and Emotional Category (F5,395 = 2.34, p<0.05) showed greater arousal in women compared to men in terms of all the negative emotion clips, including Sadness, Compassion, and Fear. However, women also reported greater arousal to Fear than to the Compassion and Sadness categories (see Fig 1B, post-hoc significance was respectively: p<0.05; p<0.01; p<0.01). Men, similarly to women, revealed greater arousal to all Emotional Categories compared to the neutral clips (all p< 0.01) and larger arousal to Erotic than to Scenery clips (p<0.01). Also, men showed greater arousal to Compassion vs Sadness but no difference was found between the Compassion and Fear clips.

### Emotional Adjectives

**Fig 2:**
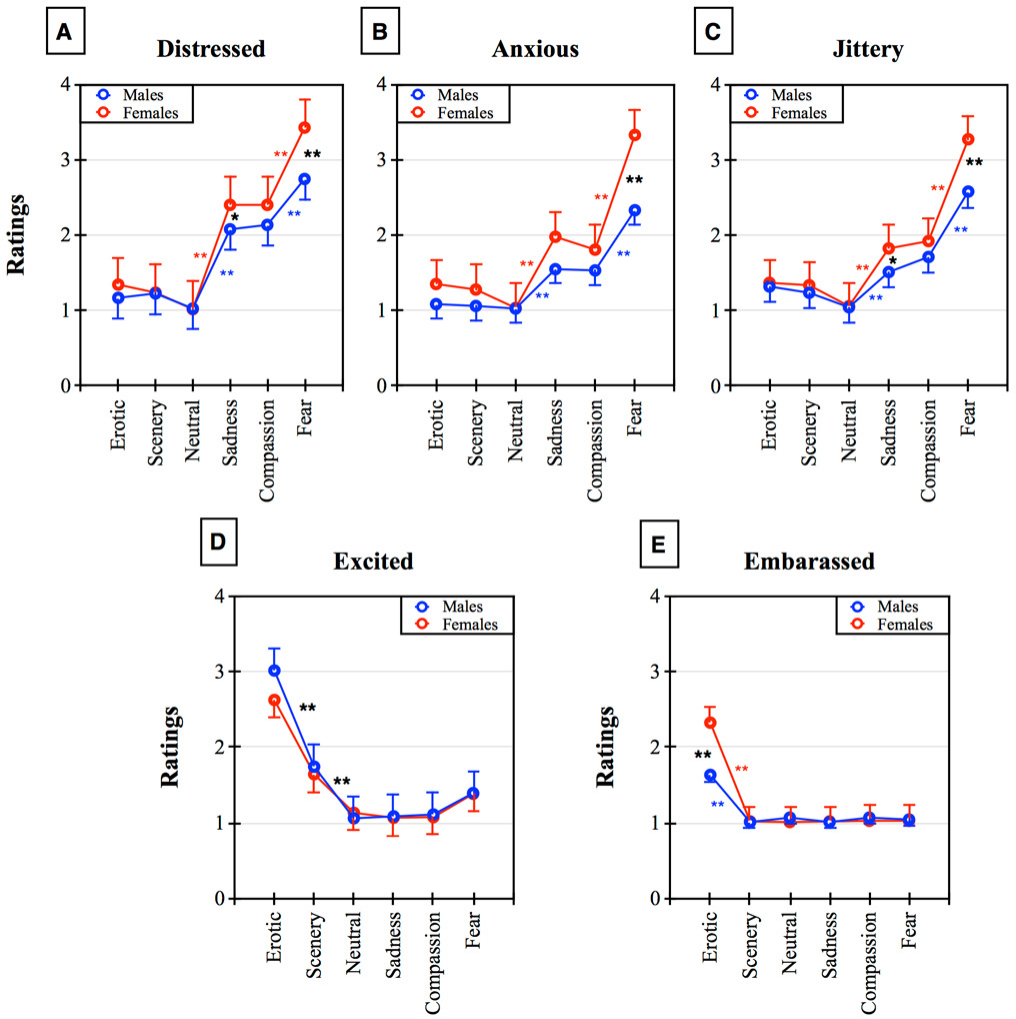
Ratings of emotional adjectives in response to the six emotional categories measured on a 1–5 analogue scale in the two genders. Panel A shows the ratings of self-perceived Distress level. Panel B shows the self-perceived Anxiety level. Panel C shows the ratings of Agitation-Jittery. Panel D shows the level of Excitement elicited by the clips. Panel E shows the self-evaluation of the Embarrassment level. Asterisks indicate * p<0.05 and ** p< 0.01. Red: within-females post-hoc comparisons. Blue: within-males post-hoc comparisons. Black: between-groups (or common within-group effects for Excited) post-hoc comparisons.

Each film clip was rated by participants according to five adjectives on a 1–5 scale (Fig 2). The most interesting results for the present study concerned the interaction between Gender and Emotional Condition. Significant interactions were found for the Distress, Anxious, and Jittery adjectives (respectively, F5,395 = 4.75, p<0.01, F5,395 = 5.88, p<0.01, and F5,395 = 5.28, p<0.01). This highlights the fact that women reported larger scores compared to men for the Fear clips (all p<0.01; Fig 2A, 2B and 2C). In addition, both genders showed significantly higher rates in all three adjectives to the Fear clips compared to other Emotional categories (all p<0.01). Also, the Sadness clips elicited higher Distress and Jittery in women compared to men (post-hoc p<0.05).

Erotic clips were rated as more exciting compared to all other categories (main effect Emotional Category F5,395 = 138.99, p<0.01, post-hoc Erotic vs. all other categories p<0.01) both by males and females (the Gender by Category interaction was not significant; Fig 2D). However, the significant two-way interaction found for the Embarrassed adjective (F5,395 = 13.06, p<0.01) revealed that women felt more embarrassed in relation to the Erotic clips compared to men (post-hoc p<0.01).

Additional analyses carried out with non-parametric tests, the Wilcoxon for paired non-repeated comparisons and the Mann-Whitney U-Test for repeated measure variables, confirmed the corresponding ANOVA post-hoc results and demonstrated the same between-group and within-group significant comparisons and effects. Only one comparison, men vs women for the Excitation adjective in relation to the Erotic condition was not significant with the ANOVA post-hoc analysis but became significant with the Mann-Whitney U-test (U = 581, p<0.05), with men showing significantly greater excitation than women.

### Correlation of Arousal with Emotional Adjective levels

Within each gender group, arousal levels have been correlated with emotional adjective levels and for each film in order to see how much arousal level explained variance within each emotional category. Results showed a cluster of significant correlations in males to unpleasant categories and Anxious, Jittery, Distressed adjectives (Table 1B), whereas women showed a similar cluster of significance, but with lower correlation values and non-significant for two adjectives (Anxious and Jittery) out three to compassion films (Table 1A). While both men and women showed a significant correlation between arousal and Excitation to pleasant Scenery an Erotic clips, males revealed a significant positive correlation also with Anxious adjective during the same pleasant films and during Neutral and Sadness categories (Table 1B). Embarrassment was the only emotional adjective not correlated in any group or film category.

**Table 1:** Within-subjects Pearson’s correlation between arousal and emotional adjectives in Females (panel A) and Males (panel B) for each film clip category.

	CORRELATION OF AROUSAL WITH EMOTIONAL ADJECTIVES				
***A(Females)***	**Anxious**	**Jittery**	**Distressed**	**Excited**	**Embarrassed**
**EROTIC**	r_39_ = .203	r_39_ = .301	r_39_ = .130	r_39_ = .762	r_39_ = .083
	n.s	n.s	n.s	p < .001	n.s
**SCENERY**	r_39_ = .288	r_39_ = .351	r_39_ = .248	r_39_ = .541	r_39_ = .153
	n.s	p < .05	n.s	p < .001	n.s
**NEUTRAL**	r_39_ = -.078	r_39_ = .119	r_39_ = -.122	r_39_ = .192	r_39_ = .048
	n.s	n.s	n.s	n.s	n.s
**SADNESS**	r_39_ = .431	r_39_ = .360	r_39_ = .396	r_39_ = .029	r_39_ = .035
	p < .01	p < .05	p < .05	n.s	n.s
**COMPASSION**	r_39_ = .232	r_39_ = .227	r_39_ = .366	r_39_ = .138	r_39_ = .020
	n.s	n.s	p < .05	n.s	n.s
**FEAR**	r_39_ = .33	r_39_ = .456	r_39_ = .495	r_39_ = .305	r_39_ = -.006
	p < .05	p < .01	p < .01	n.s	n.s
***B(Males)***	**Anxious**	**Jittery**	**Distressed**	**Excited**	**Embarrassed**
**EROTIC**	r_39_ = .362	r_39_ = .243	r_39_ = .219	r_39_ = .681	r_39_ = .289
	< .05	n.s	n.s	< .001	n.s
**SCENERY**	r_39_ = .382	r_39_ = .253	r_39_ = .291	r_39_ = .611	r_39_ = -.262
	< .05	n.s	n.s	< .001	n.s
**NEUTRAL**	r_39_ = .041	r_39_ = -.150	r_39_ = -.165	r_39_ = .473	-r_39_ = .166
	n.s	n.s	n.s	< .01	n.s
**SADNESS**	r_39_ = .451	r_39_ = .379	r_39_ = .362	r_39_ = .327	r_39_ = -.077
	< .01	< .05	< .05	< .05	n.s
**COMPASSION**	r_39_ = .404	r_39_ = .562	r_39_ = .487	r_39_ = .097	r_39_ = -.078
	< .01	< .001	< .001	n.s	n.s
**FEAR**	r_39_ = .621	r_39_ = .508	r_39_ = .505	r_39_ = .223	r_39_ = .129
	< .001	< .001	< .001	n.s	n.s

## Discussion

The present study sought to investigate sex differences in affective evaluation of six different categories of emotional clips. Although many gender studies have investigated the same categories, research has been fragmented by the lack of a control/measure of arousal level, the use of different means (slides vs. films), the comparison of only 2–3 of these stimuli within one study, and by the use of Pleasant, Neutral, and Unpleasant macro-categories, in which emotional contents had a large heterogeneity across studies. Since some categories (Scenery, Sadness, and Compassion) may need time to elicit an emotion, gender experiments can benefit from the use of film clips rather than slides. Starting from pleasant stimuli, the Erotic clips were judged highly pleasant, exciting, and arousing by both males and females. This is in line with past literature [11,22, 23, 35] and with the biological relevance of these stimuli. Within this general effect, women found Erotic clips less pleasant than the Scenery clips and this was probably related to the greater embarassment felt by women with respect to men, an effect sometimes reported from our as well as from other labs working on emotions, but that has been measured so far only by Costa and collegues [36]. Embarassement is probably a socially mediated response that decreases in non-social conditions; nevertheless, when subjects are alone in the laboratory, it can still be observed. However, in this self-evaluative paradigm, males also reported more embarassment to erotic stimuli than to other forms of stimuli, although to a minor extent (Fig 2E). Scenery clips have been used in past research (sometimes with the label “natural landscapes”) as a neutral condition [10,37,38]. Instead, as we originally hypothesized, Scenery clips were reported as being as pleasant as the erotic ones but were slightly less arousing than the Erotic and Fear clips, probably because they elicit a passive, relaxing, or contemplative state compared to more biologically relevant stimuli (e.g. Erotic and Fear), an effect that is similar in both sexes. Neutral clips that represented urban documentary proved to be effective control stimuli, with mean pleasantness ratings, significantly lower arousal compared to the other categories, no gender effects, and minimum evaluation in terms of adjectives. It is worth highlighting that in the present study, landscapes and wilderness clips were reported as being very pleasant and arousing; therefore, nature and scenery clips may be seen as problematic confounders when included in the Neutral condition (as suggested by Rottenberg and collegues [18]) but also represent a new and interesting positive emotional category.

Unpleasant clips included two very similar contents, Sadness and Compassion. In past literature, Compassion has only been reported using the terms “Sad” or “Sadness”: indeed, in past experiments, sadness was the label associated with touching, teary clips. We hypothesized that teary clips elicit a strong automatic empathic response aimed to support and help the individual who is in tears. Thus, we used the term Compassion for clips featuring tears, and used Sadness to indicate the clips that elicited sorrow without tears. Results showed that although the two unpleasant categories received similar judgements in all dependent variables, Compassion was, in both genders, more arousing than Sadness (Fig 1B). This is in line with the view that this category activates automatic empathic responses that are biologically relevant [39,25]. However, women were more sensitive to these categories, as they rated them as being more unpleasant than the neutral ones and less unpleasant than Fear. Furthermore, both categories elicited more arousal in women than men (Fig 1B). This is in agreement with the current general view that women are more responsive/vulnerable to aversive stressful conditions [3,6–8]. More Distress and Jittery adjectives were reported by women compared to men in relation to the Sadness films. This result indicates that Compassion is more activating but also inspires prosocial attitudes and empathic sharing, which dampens the feeling of sadness, while Sadness clips exacerbate loneliness and isolation and elicit more angst in women. All unpleasant categories induced more Distress, Anxiety, and Jittery than positive and neutral clips, and this occurred similarly in the two genders. The Fear clips were the most arousing and unpleasant among all the categories. In addition, women were sytematically more responsive and sensitive to Fear than men on all the scales: Unpleasantness, Arousal, Distress, Jittery, and Anxiety. This fits with the literature showing the biological relevance of defensive responses to danger/fearful stimuli but also to gender differences in the defense response [8,11,40]. Interestingly, women’s valence judgements were differentiated in almost all categories; only Sadness and Compassion were equally rated. Similarly, this greater emotional discrimination ability compared to men was observed in all the other scales. This outcome is in line with a previous review of the literature, pointing to the greater ability of women to recognize emotions compared to men [41]. Instead, men had flat valence judgements for all unpleasant clips with ratings that did not differ from the neutral ones. At first glance, men seemed to be highly insensitive to unpleasant material; however, the Arousal scale, together with the Distressed, Anxious, and Jittery scales indicate that undoubtedly men respond and discriminate negative stimuli, although seemingly to a lesser extent than women. During hominids evolution, males’ preeminent hunting and protecting roles may have made them relatively less sensitive and scared by aversive stimuli, and this might have shaped their brains accordingly [42]. However, the surprisingly flat emotional valence displayed by men to unpleasant clips, unparalleled by the other scales, points to a possible social influence and desirability in this scale: men might be willing to display insensitivity to unpleasant clips to accomplish their gender setereotyped role. Indeed, correlation analysis supports this interpretation.

In emotion experiments, arousal rather than valence, may be considered the main variable able to explain good part of variance of other quantitative variables such as anxiety, jittery, excitation, distress. Greater levels of these feelings and sensations are expected to increase with increasing arousal levels selectively for specific emotion categories. For this reason, only correlation with arousal was investigated. Correlations of emotional adjective estimation with arousal levels across the six categories of film stimuli, showed in several instances a coherent pattern, e.g. with larger correlations between arousal and Anxiety, Jittery, Distress levels for the three unpleasant clips in both males and females. Apparently in contrast with ANOVA data, in which men found compassion, sadness and fear as much unpleasant as the neutral ones and less arousing than in females, they evidenced more correlations and often to a greater extent with respect to women. We explain this as a “roof” effect in women responses, especially to aversive clips, the flattening of their arousal levels towards the highest scores reduced variance and therefore the correlations, whereas men had arousal shifted towards lower levels, thus they exhibited largest variances. We think that men, following their gender social sterotype, automatically tended to report a reduced perceived emotional impact, nevertheless the high correlations found to fear and compassion clips (especially the significant correlations with Anxiuos and Jittery levels) points to a high emotional reactivity in part of the male sample: those who perceived relatively more arousal to fear and compassion were also more anxious and agitated. Men showed also two significant correlations lacking in women, including Anxious and Jittery adjectives during compassion films, a result indicating a relatively hidden but real sensitivity of males to compassion. Unexpectedly, unlike women, males had significant correlations of arousal, during pleasant films (Scenery and Erotic), associated to Anxious adjective, and during Sadness and Neutral clips associated to Excited adjective. Concerning excitation, men’s verbal reports at the end of the session suggest that those who reported greater arousal referred greater interest and aesthetic appreciation for both Sadness and the Neutral clips (which included conventional documentaries on Paris, London, New York cities, that nevertheless have been found relatively interesting by some male participants).

In conclusion, emotional evaluation of an arousal-controlled sample of film clips of 6 different categories allowed us to add to the current knowledge about gender differences in emotional response to new categorization of emotional contents (e.g. Scenery, Sadness, Compassion) often confounded in the literature. The results point to a greater sensitivity of women to all unpleasant categories but also to their greater ability to differentiate among negative emotions with respect to men. Although the social influence of the reported effects are clearly important in some conditions, such as embarrassment during the Erotic clips, in the present setting using subjective measures, the biological compared to the environmental influences, and their interactions, could not be easily disentengled. However, the adopted experimental approach, put in a perspective focused on gender emotional differences, clearly suggests the advantage of selecting more homogenous categories of emotional film clips and implementing specific adjective-based emotional scales. Indeed, compared to past research, in the present study neutral clips were not a mix of scenery and urban landscapes; the sadness clips were not a mix of crying characters and sad situations; fear clips did not include blood and mutilations that typically induce more disgust than fear [43,44]. This strategy, together with the relative balancing of arousal level across emotional categories helped to detect more clearcut gender-related differences. Limits of the present research are the lack of personality traits measures, such as anxiety, impulsivity, neuroticism, etc. which may underlie to part of the observed gender differences, the control of sexual orientation of the sample (e.g. for homosexuals, sexual couples in the Erotic clips are not the preferred erotic stimuli) and of the menstrual phase of women’ group. Future experiments are expected to overcome the above listed limits and will study how subjective measures relate and integrate with peripheral and central psychophysiological indices across several emotional category clips.

## Supporting Information

S1 File List of the film clips with title, onset time and duration. (DOCX)

S2 File Raw data used for statistics and figures within the manuscript. (DOCX)
