# Supplementary material for: Sex Differences in Emotional Evaluation of Film Clips: Interaction with Five High Arousal Emotional Categories
Source: PLoS One. 2015 Dec 30;10(12):e0145562. doi: 10.1371/journal.pone.0145562 (PMC4696842; doi:10.1371/journal.pone.0145562)
Supplement: S1 File — (DOCX) [file pone.0145562.s001.docx]

**S1 File** – List of the film clips with title, onset time and duration

| CATEGORY | MOVIE TITLE | STARTING TIME (h/min/sec) | DURATION (min/sec) |
| --- | --- | --- | --- |
| EROTIC | *Lust* | 00:21:18 | 02:01 |
| EROTIC | *The Notebook* | 01:18:00 | 02:01 |
| EROTIC | *Monster’s Ball* | 01:13:54 | 02:01 |
| SCENERY | BBC Planet Earth’s *Mountains* | Several excerpts depicting mountainous landscapes | 01:58 |
| SCENERY | BBC Planet Earth’s *Fresh Waters* | Several excerpts depicting landscapes of rivers and waterfalls | 02:05 |
| SCENERY | BBC *Great Barrier Reef – Reef to Rainforest* | 00:00:09 | 01:55 |
| NEUTRAL | *Globe Trekker: London* | 00:10:25 | 01:56 |
| NEUTRAL | *Globe Trekker: Paris* | 00:19:29 | 02:07 |
| NEUTRAL | *Globe Trekker: New York* | 00:01:47 | 01:52 |
| SADNESS | *The Road* | 00:03:04 | 02:06 |
| SADNESS | *Blood Diamond* | 02:06:48 | 02:09 |
| SADNESS | *K-19* | 01:10:43 | 02:10 |
| COMPASSION | *The Pursuit of Happiness* | 01:26:18 | 01:58 |
| COMPASSION | *Armageddon* | 02:12:52 | 02:10 |
| COMPASSION | *Lost (Tv Series – S06E14)* | 00:34:44 | 02:10 |
| FEAR | *The Sixth Sense* | 00:51:22 | 02:10 |
| FEAR | *The Silence of the Lambs* | 01:47:02 | 02:09 |
| FEAR | *Gothika* | 00:48:19 | 02:03 |
