# Supplementary material for: Sex Differences in Emotional Evaluation of Film Clips: Interaction with Five High Arousal Emotional Categories
Source: PLoS One. 2015 Dec 30;10(12):e0145562. doi: 10.1371/journal.pone.0145562 (PMC4696842; doi:10.1371/journal.pone.0145562)
Supplement: S2 File — (DOCX) [file pone.0145562.s002.docx]

**S2 Table A**: Raw data of “Valence” ratings used for statistics and figures within the manuscript

| **Participant** | **Sex** | **Erotic** | **Scenery** | **Neutral** | **Sadness** | **Compassion** | **Fear** |
| --- | --- | --- | --- | --- | --- | --- | --- |
| 1 | F | 6.667 | 7.667 | 5.000 | 7.000 | 7.000 | 6.000 |
| 2 | M | 6.667 | 5.333 | 5.333 | 2.000 | 2.000 | 2.667 |
| 3 | M | 7.333 | 7.333 | 5.000 | 5.333 | 5.000 | 5.333 |
| 4 | F | 5.333 | 8.333 | 7.333 | 4.333 | 4.667 | 3.000 |
| 5 | M | 5.000 | 5.000 | 5.667 | 4.667 | 4.000 | 3.333 |
| 6 | F | 5.333 | 4.667 | 5.000 | 4.667 | 5.667 | 2.667 |
| 7 | M | 7.333 | 5.333 | 3.000 | 5.000 | 6.000 | 4.667 |
| 8 | M | 7.000 | 6.667 | 3.333 | 5.000 | 7.000 | 6.333 |
| 9 | F | 8.000 | 8.333 | 6.667 | 5.333 | 6.333 | 3.000 |
| 10 | M | 8.000 | 6.000 | 5.333 | 4.000 | 3.000 | 1.667 |
| 11 | F | 7.000 | 5.667 | 6.333 | 3.000 | 1.333 | 4.000 |
| 12 | F | 4.667 | 7.333 | 5.667 | 3.000 | 4.667 | 3.667 |
| 13 | F | 4.667 | 7.667 | 5.000 | 3.333 | 2.333 | 1.000 |
| 14 | M | 6.333 | 9.000 | 6.333 | 5.333 | 3.667 | 4.333 |
| 15 | F | 7.000 | 9.000 | 6.333 | 2.667 | 2.667 | 1.667 |
| 16 | M | 6.182 | 6.333 | 5.000 | 3.333 | 4.000 | 2.667 |
| 17 | F | 5.000 | 5.333 | 5.667 | 5.000 | 6.000 | 2.667 |
| 18 | M | 7.333 | 5.333 | 2.667 | 2.667 | 3.667 | 3.000 |
| 19 | F | 7.000 | 6.333 | 5.000 | 3.667 | 3.000 | 3.667 |
| 20 | M | 6.333 | 7.333 | 6.000 | 5.667 | 6.333 | 6.000 |
| 21 | F | 6.333 | 6.000 | 6.000 | 3.000 | 3.000 | 2.667 |
| 22 | M | 7.000 | 7.667 | 7.667 | 4.000 | 4.667 | 3.333 |
| 23 | F | 4.667 | 6.333 | 5.000 | 3.667 | 3.333 | 3.333 |
| 24 | M | 8.000 | 6.000 | 5.333 | 3.333 | 2.333 | 2.000 |
| 25 | F | 6.667 | 6.333 | 6.333 | 7.333 | 7.333 | 4.667 |
| 26 | M | 5.000 | 6.667 | 5.333 | 5.667 | 5.333 | 5.000 |
| 27 | F | 7.667 | 8.333 | 3.333 | 4.333 | 3.000 | 4.333 |
| 28 | M | 7.000 | 6.000 | 5.667 | 4.333 | 3.667 | 7.333 |
| 29 | F | 7.667 | 7.667 | 5.333 | 3.667 | 1.667 | 2.333 |
| 30 | M | 7.667 | 7.667 | 6.667 | 5.667 | 4.333 | 3.333 |
| 31 | F | 7.000 | 7.667 | 4.667 | 5.333 | 8.000 | 4.000 |
| 32 | M | 6.667 | 7.667 | 5.667 | 3.667 | 2.333 | 3.000 |
| 33 | F | 5.000 | 5.667 | 5.000 | 5.333 | 5.000 | 4.333 |
| 34 | F | 5.333 | 6.000 | 3.667 | 3.667 | 2.667 | 5.000 |
| 35 | F | 5.667 | 6.333 | 7.000 | 5.667 | 4.667 | 4.667 |
| 36 | F | 4.667 | 6.667 | 5.000 | 5.000 | 4.000 | 5.333 |
| 37 | F | 4.333 | 5.667 | 5.000 | 3.667 | 5.667 | 2.333 |
| 38 | F | 6.000 | 8.333 | 2.667 | 4.667 | 7.000 | 3.667 |
| 39 | M | 6.000 | 6.000 | 5.333 | 4.667 | 6.333 | 4.000 |
| 40 | M | 6.000 | 5.667 | 6.000 | 5.667 | 5.667 | 4.667 |
| 41 | M | 6.333 | 4.667 | 6.333 | 5.000 | 4.000 | 4.000 |
| 42 | M | 6.000 | 5.667 | 4.000 | 4.000 | 6.000 | 5.333 |
| 43 | M | 5.000 | 5.667 | 4.333 | 4.667 | 4.333 | 2.667 |
| 44 | M | 6.333 | 6.667 | 4.667 | 5.667 | 3.667 | 3.000 |
| 45 | F | 6.333 | 4.000 | 6.000 | 6.667 | 5.667 | 4.667 |
| 46 | F | 5.000 | 7.667 | 5.000 | 3.667 | 3.333 | 1.000 |
| 47 | F | 5.000 | 8.333 | 7.000 | 3.000 | 3.333 | 3.333 |
| 48 | F | 5.333 | 8.667 | 5.000 | 2.000 | 3.333 | 2.667 |
| 49 | F | 3.333 | 6.667 | 3.333 | 7.333 | 7.667 | 5.000 |
| 50 | F | 5.333 | 7.333 | 5.000 | 3.333 | 5.333 | 3.000 |
| 51 | F | 7.000 | 7.000 | 5.667 | 3.000 | 3.333 | 1.667 |
| 52 | F | 5.333 | 6.667 | 5.000 | 4.333 | 4.333 | 2.000 |
| 53 | F | 7.333 | 7.333 | 6.333 | 3.667 | 5.000 | 4.000 |
| 54 | F | 3.667 | 5.667 | 4.667 | 5.333 | 5.333 | 4.000 |
| 55 | F | 7.667 | 8.333 | 5.000 | 4.333 | 5.333 | 2.000 |
| 56 | F | 6.000 | 8.333 | 5.333 | 3.333 | 1.667 | 3.000 |
| 57 | F | 6.333 | 6.333 | 5.333 | 4.667 | 4.000 | 2.000 |
| 58 | F | 6.333 | 5.333 | 6.000 | 8.000 | 8.000 | 6.667 |
| 59 | F | 6.000 | 6.333 | 5.000 | 5.333 | 5.000 | 5.333 |
| 60 | F | 5.333 | 5.667 | 4.000 | 2.000 | 4.000 | 1.333 |
| 61 | F | 5.667 | 7.667 | 5.333 | 2.333 | 2.000 | 1.667 |
| 62 | F | 4.667 | 7.000 | 4.000 | 3.333 | 3.333 | 2.333 |
| 63 | F | 6.000 | 8.000 | 5.000 | 5.000 | 3.333 | 1.333 |
| 64 | M | 6.667 | 5.667 | 4.667 | 5.333 | 4.333 | 4.333 |
| 65 | M | 7.667 | 7.000 | 5.000 | 4.667 | 4.667 | 5.333 |
| 66 | M | 5.333 | 7.667 | 4.333 | 7.000 | 6.667 | 6.000 |
| 67 | M | 7.000 | 9.000 | 6.667 | 6.667 | 6.000 | 5.667 |
| 68 | M | 7.000 | 8.000 | 4.000 | 4.667 | 2.667 | 2.667 |
| 69 | M | 6.000 | 8.000 | 4.000 | 4.000 | 3.000 | 3.667 |
| 70 | M | 5.667 | 5.333 | 4.667 | 5.000 | 5.333 | 3.667 |
| 71 | M | 7.000 | 6.667 | 5.000 | 2.667 | 3.667 | 2.333 |
| 72 | M | 6.333 | 9.000 | 5.000 | 3.667 | 3.000 | 1.667 |
| 73 | M | 7.667 | 9.000 | 3.333 | 4.333 | 7.333 | 6.333 |
| 74 | M | 3.000 | 6.333 | 3.333 | 3.667 | 3.000 | 3.000 |
| 75 | M | 4.000 | 6.667 | 5.000 | 3.000 | 5.000 | 3.667 |
| 76 | M | 5.000 | 6.333 | 5.000 | 3.667 | 3.333 | 3.000 |
| 77 | M | 7.667 | 5.333 | 2.333 | 7.000 | 5.333 | 7.667 |
| 78 | M | 5.333 | 8.000 | 2.000 | 6.667 | 5.667 | 6.000 |
| 79 | M | 6.333 | 5.000 | 6.333 | 4.333 | 5.667 | 3.333 |
| 80 | M | 5.667 | 9.000 | 5.000 | 5.667 | 4.000 | 4.667 |
| 81 | M | 7.333 | 7.667 | 3.667 | 3.667 | 2.000 | 3.667 |

**S2 Table B**: Raw data of “Arousal” ratings used for statistics and figures within the manuscript

| **Participant** | **Sex** | **Erotic** | **Scenery** | **Neutral** | **Sadness** | **Compassion** | **Fear** |
| --- | --- | --- | --- | --- | --- | --- | --- |
| 1 | F | 7.000 | 5.000 | 2.667 | 5.667 | 7.000 | 6.667 |
| 2 | M | 6.333 | 3.000 | 1.333 | 4.667 | 6.333 | 4.333 |
| 3 | M | 7.333 | 6.000 | 2.333 | 6.000 | 7.667 | 5.333 |
| 4 | F | 6.333 | 2.667 | 3.000 | 5.333 | 7.333 | 6.667 |
| 5 | M | 6.000 | 3.000 | 3.000 | 4.333 | 6.000 | 7.000 |
| 6 | F | 7.000 | 3.000 | 2.667 | 5.667 | 6.333 | 6.333 |
| 7 | M | 6.333 | 3.000 | 1.333 | 3.667 | 5.000 | 5.667 |
| 8 | M | 7.000 | 5.333 | 3.000 | 5.333 | 6.333 | 6.333 |
| 9 | F | 8.667 | 6.667 | 1.667 | 5.667 | 7.667 | 8.333 |
| 10 | M | 7.667 | 4.000 | 1.000 | 6.667 | 8.333 | 8.333 |
| 11 | F | 7.333 | 8.333 | 4.667 | 7.667 | 8.333 | 8.000 |
| 12 | F | 6.333 | 6.333 | 4.667 | 6.667 | 7.333 | 6.333 |
| 13 | F | 7.333 | 5.667 | 4.667 | 5.000 | 6.000 | 9.000 |
| 14 | M | 4.667 | 3.000 | 2.000 | 2.667 | 3.333 | 2.667 |
| 15 | F | 7.333 | 6.000 | 5.333 | 6.667 | 7.333 | 8.000 |
| 16 | M | 4.667 | 2.333 | 1.333 | 2.333 | 3.000 | 4.667 |
| 17 | F | 6.667 | 3.000 | 3.667 | 5.000 | 7.000 | 8.000 |
| 18 | M | 7.000 | 4.667 | 2.000 | 4.667 | 5.667 | 6.333 |
| 19 | F | 6.667 | 5.667 | 5.000 | 4.667 | 6.667 | 7.000 |
| 20 | M | 4.000 | 4.333 | 2.000 | 5.333 | 4.667 | 4.000 |
| 21 | F | 6.667 | 5.333 | 4.667 | 6.000 | 7.000 | 8.000 |
| 22 | M | 7.667 | 7.667 | 6.667 | 6.333 | 6.333 | 6.667 |
| 23 | F | 4.333 | 6.000 | 3.000 | 5.333 | 7.000 | 6.667 |
| 24 | M | 8.000 | 4.333 | 1.667 | 3.333 | 5.667 | 7.000 |
| 25 | F | 7.000 | 3.000 | 2.667 | 5.333 | 7.333 | 8.000 |
| 26 | M | 3.000 | 2.667 | 1.667 | 1.333 | 2.333 | 2.333 |
| 27 | F | 8.333 | 7.667 | 1.000 | 6.667 | 5.000 | 7.667 |
| 28 | M | 6.333 | 5.333 | 1.333 | 4.000 | 5.667 | 6.667 |
| 29 | F | 7.333 | 6.000 | 2.000 | 8.333 | 9.000 | 8.667 |
| 30 | M | 6.667 | 6.333 | 4.000 | 3.333 | 3.000 | 6.667 |
| 31 | F | 6.333 | 5.333 | 3.333 | 4.667 | 6.333 | 7.333 |
| 32 | M | 6.333 | 5.667 | 1.333 | 3.333 | 5.000 | 6.333 |
| 33 | F | 6.000 | 5.667 | 3.333 | 5.667 | 6.333 | 6.000 |
| 34 | F | 5.333 | 4.333 | 3.000 | 6.000 | 7.333 | 6.667 |
| 35 | F | 5.667 | 6.000 | 6.000 | 5.667 | 6.000 | 5.667 |
| 36 | F | 8.000 | 3.667 | 1.667 | 5.667 | 7.333 | 6.333 |
| 37 | F | 8.000 | 2.333 | 1.000 | 6.667 | 7.667 | 8.000 |
| 38 | F | 5.667 | 6.667 | 1.000 | 2.333 | 4.333 | 7.333 |
| 39 | M | 3.667 | 3.667 | 1.667 | 2.667 | 3.667 | 4.000 |
| 40 | M | 6.000 | 6.000 | 6.000 | 6.000 | 6.667 | 6.667 |
| 41 | M | 7.667 | 3.667 | 4.667 | 2.000 | 3.667 | 5.000 |
| 42 | M | 6.333 | 4.000 | 1.667 | 3.667 | 5.333 | 6.000 |
| 43 | M | 5.667 | 3.667 | 1.333 | 2.667 | 4.333 | 5.333 |
| 44 | M | 7.333 | 5.667 | 2.000 | 6.000 | 5.667 | 6.667 |
| 45 | F | 6.333 | 3.000 | 3.667 | 5.667 | 5.000 | 6.667 |
| 46 | F | 4.000 | 6.000 | 3.000 | 6.333 | 7.333 | 8.000 |
| 47 | F | 6.000 | 5.333 | 1.667 | 4.000 | 4.333 | 7.667 |
| 48 | F | 5.667 | 8.667 | 5.000 | 7.333 | 8.667 | 8.000 |
| 49 | F | 3.000 | 5.000 | 1.333 | 8.333 | 7.333 | 7.667 |
| 50 | F | 5.333 | 5.333 | 2.333 | 4.667 | 5.667 | 5.667 |
| 51 | F | 7.000 | 7.000 | 3.333 | 3.667 | 4.667 | 8.000 |
| 52 | F | 6.667 | 6.667 | 2.333 | 7.667 | 7.000 | 6.667 |
| 53 | F | 6.333 | 5.667 | 3.333 | 4.667 | 5.667 | 7.000 |
| 54 | F | 5.000 | 5.333 | 2.000 | 6.000 | 7.000 | 7.000 |
| 55 | F | 7.667 | 4.667 | 1.000 | 5.000 | 5.667 | 7.000 |
| 56 | F | 6.333 | 7.000 | 2.000 | 5.333 | 7.667 | 7.000 |
| 57 | F | 6.333 | 6.333 | 4.000 | 6.000 | 7.000 | 6.667 |
| 58 | F | 8.667 | 3.667 | 5.000 | 8.000 | 8.333 | 8.667 |
| 59 | F | 5.000 | 3.667 | 2.667 | 4.333 | 4.333 | 5.667 |
| 60 | F | 7.667 | 7.667 | 1.000 | 7.000 | 5.667 | 9.000 |
| 61 | F | 7.000 | 6.667 | 1.333 | 6.000 | 7.667 | 7.667 |
| 62 | F | 7.000 | 7.333 | 1.667 | 6.000 | 6.000 | 6.333 |
| 63 | F | 6.333 | 5.667 | 2.000 | 5.667 | 7.667 | 6.000 |
| 64 | M | 6.667 | 6.333 | 2.333 | 6.667 | 6.667 | 7.333 |
| 65 | M | 7.667 | 4.333 | 1.667 | 4.000 | 3.667 | 7.000 |
| 66 | M | 6.000 | 7.333 | 1.667 | 7.000 | 5.000 | 6.333 |
| 67 | M | 6.333 | 8.667 | 4.667 | 6.000 | 5.000 | 5.000 |
| 68 | M | 6.333 | 8.000 | 3.000 | 7.667 | 8.000 | 7.667 |
| 69 | M | 7.000 | 7.667 | 1.000 | 6.667 | 7.667 | 7.000 |
| 70 | M | 7.000 | 2.333 | 2.000 | 2.000 | 4.667 | 5.667 |
| 71 | M | 6.667 | 4.333 | 2.000 | 4.667 | 6.000 | 5.667 |
| 72 | M | 7.333 | 9.000 | 5.333 | 7.667 | 9.000 | 7.667 |
| 73 | M | 7.333 | 8.333 | 3.333 | 4.000 | 7.667 | 6.667 |
| 74 | M | 6.000 | 6.000 | 3.333 | 4.667 | 6.667 | 4.667 |
| 75 | M | 5.667 | 5.667 | 2.000 | 6.000 | 5.333 | 6.667 |
| 76 | M | 5.000 | 3.333 | 1.000 | 5.000 | 3.333 | 6.000 |
| 77 | M | 7.333 | 4.333 | 1.333 | 7.000 | 7.333 | 8.667 |
| 78 | M | 4.000 | 7.667 | 1.000 | 7.333 | 5.667 | 5.667 |
| 79 | M | 6.333 | 2.000 | 5.000 | 8.000 | 7.333 | 6.333 |
| 80 | M | 5.333 | 5.000 | 2.667 | 5.333 | 6.333 | 5.667 |
| 81 | M | 6.333 | 6.000 | 1.000 | 4.000 | 5.667 | 7.333 |

**S2 Table C**: Raw data of “Distressed” ratings used for statistics and figures within the manuscript

| **Participant** | **Sex** | **Erotic** | **Scenery** | **Neutral** | **Sadness** | **Compassion** | **Fear** |
| --- | --- | --- | --- | --- | --- | --- | --- |
| 1 | F | 1.333 | 1.000 | 1.000 | 1.000 | 1.333 | 1.667 |
| 2 | M | 1.000 | 1.000 | 1.000 | 4.000 | 3.667 | 3.333 |
| 3 | M | 1.667 | 1.000 | 1.000 | 2.000 | 3.000 | 2.333 |
| 4 | F | 1.000 | 1.333 | 1.000 | 1.667 | 1.333 | 2.667 |
| 5 | M | 1.667 | 1.333 | 1.000 | 1.667 | 2.333 | 3.000 |
| 6 | F | 1.000 | 1.000 | 1.000 | 2.333 | 2.667 | 3.333 |
| 7 | M | 1.000 | 1.000 | 1.333 | 2.667 | 2.667 | 3.000 |
| 8 | M | 1.000 | 1.667 | 1.000 | 1.667 | 1.000 | 2.667 |
| 9 | F | 1.000 | 1.667 | 1.000 | 3.000 | 3.000 | 4.667 |
| 10 | M | 1.667 | 1.000 | 1.000 | 3.000 | 2.333 | 4.333 |
| 11 | F | 1.667 | 2.333 | 1.000 | 4.333 | 4.333 | 3.333 |
| 12 | F | 1.333 | 1.000 | 1.000 | 3.000 | 2.667 | 3.333 |
| 13 | F | 1.667 | 1.333 | 1.000 | 1.667 | 1.667 | 4.000 |
| 14 | M | 1.000 | 1.000 | 1.000 | 2.333 | 2.000 | 2.333 |
| 15 | F | 1.333 | 1.000 | 1.000 | 3.000 | 2.667 | 4.667 |
| 16 | M | 1.000 | 1.000 | 1.000 | 1.667 | 1.333 | 2.333 |
| 17 | F | 1.000 | 1.000 | 1.000 | 1.667 | 3.000 | 3.333 |
| 18 | M | 1.000 | 1.000 | 1.000 | 2.667 | 2.667 | 2.333 |
| 19 | F | 1.000 | 1.000 | 1.000 | 2.333 | 2.667 | 4.000 |
| 20 | M | 1.000 | 1.000 | 1.000 | 1.667 | 1.000 | 1.667 |
| 21 | F | 1.000 | 1.333 | 1.000 | 1.667 | 2.333 | 4.000 |
| 22 | M | 1.667 | 2.000 | 1.000 | 2.000 | 2.333 | 4.000 |
| 23 | F | 1.000 | 1.333 | 1.000 | 2.333 | 2.667 | 3.000 |
| 24 | M | 1.333 | 1.000 | 1.000 | 2.667 | 3.000 | 3.000 |
| 25 | F | 1.333 | 1.333 | 1.000 | 2.333 | 1.667 | 4.000 |
| 26 | M | 1.000 | 1.000 | 1.000 | 1.000 | 1.000 | 1.000 |
| 27 | F | 1.667 | 1.000 | 1.333 | 3.000 | 2.667 | 2.667 |
| 28 | M | 1.000 | 3.000 | 1.000 | 2.000 | 1.667 | 1.333 |
| 29 | F | 1.333 | 1.000 | 1.000 | 2.000 | 3.000 | 2.667 |
| 30 | M | 1.333 | 2.000 | 1.000 | 2.000 | 1.667 | 3.000 |
| 31 | F | 1.000 | 1.000 | 1.000 | 1.667 | 2.333 | 2.333 |
| 32 | M | 1.000 | 1.000 | 1.000 | 1.667 | 1.667 | 2.667 |
| 33 | F | 1.000 | 1.000 | 1.000 | 1.000 | 1.000 | 3.000 |
| 34 | F | 1.000 | 1.000 | 1.000 | 3.000 | 2.667 | 2.000 |
| 35 | F | 1.000 | 1.000 | 1.000 | 2.000 | 2.000 | 2.333 |
| 36 | F | 1.667 | 1.000 | 1.000 | 2.667 | 2.667 | 1.667 |
| 37 | F | 2.000 | 1.667 | 1.000 | 3.667 | 3.333 | 4.000 |
| 38 | F | 1.667 | 1.000 | 1.000 | 2.000 | 1.667 | 4.000 |
| 39 | M | 1.667 | 1.000 | 1.000 | 1.667 | 1.000 | 3.000 |
| 40 | M | 1.000 | 1.000 | 1.000 | 2.667 | 2.000 | 2.667 |
| 41 | M | 1.000 | 1.000 | 1.000 | 1.000 | 2.000 | 2.000 |
| 42 | M | 1.333 | 1.000 | 1.000 | 1.333 | 1.333 | 1.333 |
| 43 | M | 1.000 | 1.333 | 1.667 | 1.667 | 2.667 | 3.667 |
| 44 | M | 1.000 | 1.333 | 1.000 | 1.333 | 1.000 | 2.667 |
| 45 | F | 1.000 | 1.000 | 1.000 | 1.667 | 1.667 | 3.667 |
| 46 | F | 1.000 | 1.333 | 1.000 | 1.333 | 1.667 | 5.000 |
| 47 | F | 1.667 | 1.000 | 1.000 | 1.667 | 2.000 | 3.000 |
| 48 | F | 1.000 | 1.000 | 1.000 | 3.667 | 2.333 | 3.333 |
| 49 | F | 2.000 | 1.000 | 1.000 | 3.333 | 3.000 | 3.667 |
| 50 | F | 1.000 | 1.000 | 1.000 | 3.000 | 2.000 | 4.000 |
| 51 | F | 2.000 | 3.667 | 1.333 | 3.000 | 2.667 | 4.333 |
| 52 | F | 2.000 | 1.000 | 1.000 | 3.000 | 2.333 | 4.000 |
| 53 | F | 1.000 | 1.333 | 1.000 | 3.667 | 3.000 | 4.333 |
| 54 | F | 2.000 | 1.000 | 1.000 | 2.000 | 2.667 | 2.667 |
| 55 | F | 1.000 | 1.000 | 1.000 | 2.333 | 2.333 | 3.667 |
| 56 | F | 2.000 | 1.333 | 1.000 | 2.333 | 3.333 | 3.333 |
| 57 | F | 1.000 | 1.000 | 1.000 | 1.667 | 2.000 | 2.667 |
| 58 | F | 1.667 | 1.333 | 1.000 | 2.667 | 2.333 | 4.000 |
| 59 | F | 1.000 | 1.000 | 1.000 | 1.000 | 1.000 | 1.667 |
| 60 | F | 1.000 | 2.333 | 1.000 | 3.667 | 3.333 | 4.667 |
| 61 | F | 2.000 | 1.000 | 1.000 | 3.000 | 3.667 | 4.333 |
| 62 | F | 1.667 | 1.000 | 1.000 | 3.000 | 2.333 | 4.333 |
| 63 | F | 1.000 | 1.000 | 1.000 | 1.000 | 1.333 | 3.000 |
| 64 | M | 1.333 | 1.333 | 1.000 | 2.333 | 2.333 | 2.667 |
| 65 | M | 1.333 | 1.000 | 1.000 | 2.000 | 2.000 | 2.000 |
| 66 | M | 1.000 | 1.000 | 1.000 | 2.000 | 1.667 | 2.000 |
| 67 | M | 1.000 | 1.000 | 1.000 | 1.667 | 1.333 | 2.333 |
| 68 | M | 1.000 | 1.333 | 1.000 | 2.667 | 3.000 | 3.333 |
| 69 | M | 1.000 | 1.000 | 1.000 | 1.667 | 1.667 | 4.000 |
| 70 | M | 1.333 | 1.000 | 1.000 | 1.333 | 2.000 | 3.000 |
| 71 | M | 1.000 | 1.333 | 1.000 | 3.000 | 3.000 | 3.667 |
| 72 | M | 1.667 | 2.000 | 1.000 | 3.667 | 4.333 | 4.333 |
| 73 | M | 1.000 | 1.000 | 1.000 | 1.333 | 1.333 | 1.667 |
| 74 | M | 1.333 | 1.000 | 1.000 | 2.667 | 3.333 | 2.333 |
| 75 | M | 1.000 | 1.000 | 1.000 | 2.000 | 1.333 | 2.667 |
| 76 | M | 1.333 | 1.000 | 1.000 | 2.333 | 2.000 | 2.667 |
| 77 | M | 1.000 | 1.000 | 1.000 | 2.000 | 3.000 | 4.000 |
| 78 | M | 1.000 | 1.000 | 1.000 | 2.000 | 2.333 | 2.000 |
| 79 | M | 1.000 | 1.000 | 1.000 | 2.333 | 3.000 | 3.333 |
| 80 | M | 1.000 | 1.000 | 1.000 | 1.000 | 1.000 | 2.333 |
| 81 | M | 1.000 | 2.333 | 1.000 | 2.667 | 3.333 | 3.667 |

**S2 Table D**: Raw data of “Embarrassed” ratings used for statistics and figures within the manuscript

| **Participant** | **Sex** | **Erotic** | **Scenery** | **Neutral** | **Sadness** | **Compassion** | **Fear** |
| --- | --- | --- | --- | --- | --- | --- | --- |
| 1 | F | 2.000 | 1.000 | 1.000 | 1.000 | 1.000 | 1.000 |
| 2 | M | 1.000 | 1.000 | 1.000 | 1.000 | 1.000 | 1.000 |
| 3 | M | 1.000 | 1.000 | 1.000 | 1.000 | 1.000 | 1.000 |
| 4 | F | 2.333 | 1.000 | 1.000 | 1.000 | 1.000 | 1.000 |
| 5 | M | 1.000 | 1.000 | 1.000 | 1.000 | 1.333 | 1.000 |
| 6 | F | 2.667 | 1.000 | 1.000 | 1.000 | 1.333 | 1.000 |
| 7 | M | 1.000 | 1.000 | 1.000 | 1.000 | 1.000 | 1.000 |
| 8 | M | 3.000 | 1.000 | 1.000 | 1.000 | 1.000 | 1.000 |
| 9 | F | 3.333 | 1.000 | 1.000 | 1.000 | 1.000 | 1.000 |
| 10 | M | 2.000 | 1.000 | 1.000 | 1.000 | 1.000 | 1.000 |
| 11 | F | 4.333 | 1.000 | 1.000 | 1.000 | 1.000 | 1.000 |
| 12 | F | 2.667 | 1.000 | 1.000 | 1.000 | 1.000 | 1.000 |
| 13 | F | 2.333 | 1.000 | 1.000 | 1.000 | 1.000 | 1.000 |
| 14 | M | 2.000 | 1.000 | 1.000 | 1.000 | 1.000 | 1.000 |
| 15 | F | 1.333 | 1.000 | 1.000 | 1.000 | 1.000 | 1.000 |
| 16 | M | 1.000 | 1.000 | 1.000 | 1.000 | 1.000 | 1.000 |
| 17 | F | 2.333 | 1.000 | 1.000 | 1.000 | 1.000 | 1.000 |
| 18 | M | 1.333 | 1.000 | 1.000 | 1.000 | 1.333 | 1.000 |
| 19 | F | 4.000 | 1.000 | 1.000 | 1.000 | 1.000 | 1.000 |
| 20 | M | 1.000 | 1.000 | 1.000 | 1.000 | 1.000 | 1.000 |
| 21 | F | 2.333 | 1.000 | 1.000 | 1.333 | 1.333 | 1.000 |
| 22 | M | 2.667 | 1.000 | 1.000 | 1.000 | 1.333 | 2.000 |
| 23 | F | 2.000 | 1.000 | 1.000 | 1.000 | 1.000 | 1.000 |
| 24 | M | 1.333 | 1.000 | 1.000 | 1.000 | 1.000 | 1.333 |
| 25 | F | 1.000 | 1.000 | 1.000 | 1.000 | 1.000 | 1.000 |
| 26 | M | 1.333 | 1.000 | 1.000 | 1.000 | 1.000 | 1.000 |
| 27 | F | 1.000 | 1.000 | 1.000 | 1.000 | 1.000 | 1.000 |
| 28 | M | 1.000 | 1.000 | 1.000 | 1.000 | 1.000 | 1.000 |
| 29 | F | 1.000 | 1.000 | 1.000 | 1.000 | 1.000 | 1.000 |
| 30 | M | 1.333 | 1.000 | 1.000 | 1.000 | 1.000 | 1.000 |
| 31 | F | 1.667 | 1.000 | 1.000 | 1.000 | 1.000 | 1.000 |
| 32 | M | 1.000 | 1.000 | 1.000 | 1.000 | 1.000 | 1.000 |
| 33 | F | 2.000 | 1.000 | 1.000 | 1.000 | 1.000 | 1.000 |
| 34 | F | 1.667 | 1.000 | 1.000 | 1.000 | 1.000 | 1.333 |
| 35 | F | 1.333 | 1.000 | 1.000 | 1.000 | 1.000 | 1.000 |
| 36 | F | 3.000 | 1.000 | 1.000 | 1.000 | 1.000 | 1.000 |
| 37 | F | 4.000 | 1.000 | 1.000 | 1.000 | 1.000 | 1.000 |
| 38 | F | 2.000 | 1.000 | 1.000 | 1.000 | 1.000 | 1.000 |
| 39 | M | 1.000 | 1.000 | 1.000 | 1.000 | 1.000 | 1.000 |
| 40 | M | 2.000 | 1.000 | 1.000 | 1.000 | 1.000 | 1.000 |
| 41 | M | 4.000 | 1.000 | 1.000 | 1.000 | 1.000 | 1.000 |
| 42 | M | 1.000 | 1.000 | 1.000 | 1.000 | 1.000 | 1.000 |
| 43 | M | 2.000 | 1.000 | 1.000 | 1.000 | 1.000 | 1.000 |
| 44 | M | 2.333 | 1.000 | 1.000 | 1.000 | 1.000 | 1.000 |
| 45 | F | 2.667 | 1.000 | 1.000 | 1.000 | 1.000 | 1.000 |
| 46 | F | 1.667 | 1.000 | 1.000 | 1.000 | 1.000 | 1.000 |
| 47 | F | 3.000 | 1.000 | 1.000 | 1.000 | 1.000 | 1.000 |
| 48 | F | 1.667 | 1.000 | 1.000 | 1.000 | 1.000 | 1.000 |
| 49 | F | 2.333 | 1.000 | 1.000 | 1.000 | 1.000 | 1.000 |
| 50 | F | 3.667 | 1.000 | 1.000 | 1.000 | 1.000 | 1.000 |
| 51 | F | 2.000 | 1.667 | 1.333 | 1.000 | 1.000 | 1.333 |
| 52 | F | 2.667 | 1.000 | 1.000 | 1.000 | 1.000 | 1.000 |
| 53 | F | 3.667 | 1.000 | 1.000 | 1.000 | 1.000 | 1.000 |
| 54 | F | 3.000 | 1.000 | 1.000 | 1.333 | 1.333 | 1.333 |
| 55 | F | 1.667 | 1.000 | 1.000 | 1.000 | 1.000 | 1.000 |
| 56 | F | 2.667 | 1.000 | 1.000 | 1.000 | 1.000 | 1.000 |
| 57 | F | 1.000 | 1.000 | 1.000 | 1.000 | 1.000 | 1.000 |
| 58 | F | 1.000 | 1.000 | 1.000 | 1.000 | 1.000 | 1.000 |
| 59 | F | 2.000 | 1.000 | 1.000 | 1.000 | 1.000 | 1.000 |
| 60 | F | 4.667 | 1.000 | 1.000 | 1.000 | 1.000 | 1.000 |
| 61 | F | 1.333 | 1.000 | 1.000 | 1.000 | 1.000 | 1.000 |
| 62 | F | 2.667 | 1.000 | 1.000 | 1.000 | 1.000 | 1.000 |
| 63 | F | 1.667 | 1.000 | 1.000 | 1.000 | 1.000 | 1.000 |
| 64 | M | 2.000 | 1.000 | 1.000 | 1.000 | 1.000 | 1.000 |
| 65 | M | 1.000 | 1.000 | 2.000 | 1.000 | 1.000 | 1.000 |
| 66 | M | 1.000 | 1.000 | 1.000 | 1.000 | 1.000 | 1.000 |
| 67 | M | 1.000 | 1.000 | 1.000 | 1.000 | 1.000 | 1.000 |
| 68 | M | 2.000 | 1.000 | 1.000 | 1.000 | 1.000 | 1.000 |
| 69 | M | 3.000 | 1.000 | 1.667 | 1.000 | 1.000 | 1.000 |
| 70 | M | 1.000 | 1.000 | 1.000 | 1.000 | 1.333 | 1.000 |
| 71 | M | 2.333 | 1.000 | 1.000 | 1.000 | 1.000 | 1.000 |
| 72 | M | 1.333 | 1.000 | 1.000 | 1.000 | 1.000 | 1.000 |
| 73 | M | 1.667 | 1.000 | 1.000 | 1.333 | 1.333 | 1.333 |
| 74 | M | 1.000 | 1.000 | 1.000 | 1.000 | 1.000 | 1.000 |
| 75 | M | 2.333 | 1.000 | 1.000 | 1.000 | 1.000 | 1.000 |
| 76 | M | 1.000 | 1.000 | 1.000 | 1.000 | 1.667 | 1.000 |
| 77 | M | 2.333 | 1.000 | 1.000 | 1.000 | 1.000 | 1.000 |
| 78 | M | 2.333 | 1.000 | 1.000 | 1.000 | 1.000 | 1.000 |
| 79 | M | 1.667 | 1.333 | 1.000 | 1.000 | 1.000 | 1.000 |
| 80 | M | 1.000 | 1.000 | 1.667 | 1.000 | 1.000 | 1.000 |
| 81 | M | 2.000 | 1.000 | 1.333 | 1.000 | 1.333 | 1.000 |

**S2 Table E**: Raw data of “Excited” ratings used for statistics and figures within the manuscript

| **Participant** | **Sex** | **Erotic** | **Scenery** | **Neutral** | **Sadness** | **Compassion** | **Fear** |
| --- | --- | --- | --- | --- | --- | --- | --- |
| 1 | F | 2.667 | 1.000 | 1.000 | 1.000 | 1.000 | 1.000 |
| 2 | M | 3.333 | 1.667 | 1.333 | 1.000 | 1.000 | 1.667 |
| 3 | M | 4.000 | 1.333 | 1.000 | 1.000 | 1.000 | 1.333 |
| 4 | F | 3.000 | 1.000 | 1.000 | 1.000 | 1.000 | 1.000 |
| 5 | M | 2.333 | 1.000 | 1.000 | 1.000 | 1.333 | 1.000 |
| 6 | F | 3.000 | 1.000 | 1.000 | 1.000 | 1.000 | 1.000 |
| 7 | M | 3.333 | 2.000 | 1.000 | 1.000 | 1.333 | 1.000 |
| 8 | M | 3.333 | 1.333 | 1.000 | 1.000 | 1.000 | 1.000 |
| 9 | F | 4.333 | 1.667 | 1.667 | 1.000 | 1.000 | 1.667 |
| 10 | M | 3.000 | 3.000 | 1.000 | 1.000 | 1.333 | 1.667 |
| 11 | F | 4.333 | 3.667 | 1.000 | 1.333 | 1.000 | 2.667 |
| 12 | F | 3.000 | 2.000 | 1.000 | 2.000 | 1.333 | 2.667 |
| 13 | F | 2.000 | 2.333 | 2.333 | 1.667 | 1.333 | 3.667 |
| 14 | M | 2.667 | 1.667 | 1.000 | 1.333 | 1.000 | 1.333 |
| 15 | F | 3.333 | 4.333 | 2.000 | 1.000 | 1.000 | 1.000 |
| 16 | M | 2.667 | 1.000 | 1.000 | 1.000 | 1.000 | 1.000 |
| 17 | F | 2.000 | 1.000 | 1.000 | 1.000 | 1.000 | 1.000 |
| 18 | M | 4.000 | 1.000 | 1.000 | 1.000 | 1.000 | 1.333 |
| 19 | F | 3.333 | 1.333 | 1.000 | 1.000 | 1.000 | 1.000 |
| 20 | M | 1.333 | 1.333 | 1.000 | 1.000 | 1.000 | 2.333 |
| 21 | F | 2.333 | 1.333 | 1.333 | 1.000 | 1.000 | 1.333 |
| 22 | M | 4.000 | 2.333 | 1.333 | 1.000 | 1.333 | 1.667 |
| 23 | F | 1.000 | 1.000 | 1.000 | 1.000 | 1.000 | 1.000 |
| 24 | M | 4.667 | 1.667 | 1.000 | 1.000 | 1.000 | 1.000 |
| 25 | F | 3.667 | 1.333 | 1.000 | 1.000 | 1.000 | 1.333 |
| 26 | M | 1.333 | 1.000 | 1.000 | 1.000 | 1.000 | 1.000 |
| 27 | F | 4.333 | 2.667 | 1.000 | 1.000 | 1.000 | 1.000 |
| 28 | M | 2.333 | 1.333 | 1.000 | 1.000 | 1.000 | 1.667 |
| 29 | F | 2.000 | 2.333 | 1.000 | 1.000 | 1.000 | 1.333 |
| 30 | M | 3.333 | 1.000 | 1.333 | 1.000 | 1.333 | 1.000 |
| 31 | F | 2.333 | 1.000 | 1.000 | 1.000 | 1.000 | 1.000 |
| 32 | M | 2.667 | 3.000 | 1.000 | 1.000 | 1.000 | 1.667 |
| 33 | F | 2.000 | 1.000 | 1.000 | 1.000 | 1.000 | 1.000 |
| 34 | F | 2.333 | 1.333 | 1.667 | 1.000 | 1.667 | 2.000 |
| 35 | F | 2.000 | 1.000 | 1.000 | 1.000 | 1.000 | 1.000 |
| 36 | F | 3.333 | 1.000 | 1.000 | 1.000 | 1.000 | 1.000 |
| 37 | F | 3.667 | 1.000 | 1.000 | 1.000 | 1.000 | 1.000 |
| 38 | F | 1.667 | 1.000 | 1.000 | 1.000 | 1.000 | 1.000 |
| 39 | M | 2.667 | 1.667 | 1.000 | 1.000 | 1.333 | 1.000 |
| 40 | M | 2.667 | 1.333 | 1.333 | 1.000 | 1.000 | 1.000 |
| 41 | M | 3.667 | 1.333 | 2.000 | 1.000 | 1.000 | 1.333 |
| 42 | M | 2.333 | 1.000 | 1.000 | 1.000 | 1.000 | 1.333 |
| 43 | M | 2.667 | 1.667 | 1.000 | 1.000 | 1.000 | 1.333 |
| 44 | M | 2.667 | 1.000 | 1.000 | 1.000 | 1.000 | 1.333 |
| 45 | F | 2.333 | 1.333 | 1.000 | 1.000 | 1.000 | 1.667 |
| 46 | F | 2.333 | 2.000 | 1.000 | 1.000 | 1.333 | 1.667 |
| 47 | F | 2.000 | 1.333 | 1.000 | 1.000 | 1.000 | 1.000 |
| 48 | F | 1.667 | 1.333 | 1.000 | 1.000 | 1.000 | 1.000 |
| 49 | F | 1.000 | 1.000 | 1.000 | 1.000 | 1.000 | 1.000 |
| 50 | F | 2.333 | 1.000 | 1.000 | 1.000 | 1.000 | 1.000 |
| 51 | F | 3.000 | 3.000 | 1.000 | 1.333 | 1.333 | 1.667 |
| 52 | F | 1.667 | 2.000 | 1.000 | 1.000 | 1.000 | 1.667 |
| 53 | F | 3.333 | 2.000 | 1.333 | 1.000 | 1.000 | 1.333 |
| 54 | F | 1.667 | 1.667 | 1.000 | 1.000 | 1.333 | 1.667 |
| 55 | F | 4.000 | 1.000 | 1.000 | 1.000 | 1.000 | 1.333 |
| 56 | F | 2.000 | 1.333 | 1.000 | 1.000 | 1.000 | 1.000 |
| 57 | F | 2.667 | 2.000 | 1.000 | 1.333 | 1.000 | 1.000 |
| 58 | F | 4.000 | 1.000 | 1.000 | 1.000 | 1.667 | 1.667 |
| 59 | F | 1.667 | 1.000 | 1.000 | 1.000 | 1.000 | 1.000 |
| 60 | F | 3.000 | 1.667 | 1.000 | 1.000 | 1.000 | 1.333 |
| 61 | F | 3.000 | 2.667 | 1.000 | 1.000 | 1.000 | 2.000 |
| 62 | F | 2.000 | 3.667 | 2.000 | 1.000 | 1.000 | 2.000 |
| 63 | F | 2.333 | 1.000 | 1.000 | 1.000 | 1.000 | 1.000 |
| 64 | M | 3.333 | 1.000 | 1.000 | 1.333 | 1.000 | 1.667 |
| 65 | M | 4.000 | 2.000 | 1.000 | 1.000 | 1.333 | 3.000 |
| 66 | M | 2.333 | 3.000 | 1.000 | 2.000 | 1.000 | 1.333 |
| 67 | M | 2.667 | 4.333 | 1.000 | 1.000 | 1.000 | 1.000 |
| 68 | M | 2.333 | 2.333 | 1.000 | 1.000 | 1.000 | 1.333 |
| 69 | M | 2.667 | 2.667 | 1.000 | 1.000 | 1.000 | 2.333 |
| 70 | M | 3.333 | 1.000 | 1.000 | 1.000 | 1.000 | 2.000 |
| 71 | M | 3.667 | 1.000 | 1.000 | 1.000 | 1.000 | 1.000 |
| 72 | M | 3.667 | 2.667 | 1.000 | 1.000 | 1.000 | 1.000 |
| 73 | M | 4.333 | 3.667 | 1.000 | 1.000 | 1.000 | 1.667 |
| 74 | M | 2.333 | 3.000 | 1.000 | 1.333 | 1.667 | 1.333 |
| 75 | M | 1.667 | 1.000 | 1.000 | 1.000 | 1.000 | 1.000 |
| 76 | M | 3.667 | 1.333 | 1.000 | 1.000 | 1.000 | 1.000 |
| 77 | M | 3.333 | 1.333 | 1.000 | 1.667 | 2.000 | 2.667 |
| 78 | M | 2.667 | 2.667 | 1.000 | 1.667 | 1.333 | 1.333 |
| 79 | M | 3.333 | 1.000 | 1.000 | 1.000 | 1.000 | 1.000 |
| 80 | M | 2.667 | 1.000 | 1.000 | 1.000 | 1.000 | 1.000 |
| 81 | M | 3.667 | 1.000 | 1.000 | 1.000 | 1.000 | 1.000 |

**S2 Table F**: Raw data of “Jittery” ratings used for statistics and figures within the manuscript

| **Participant** | **Sex** | **Erotic** | **Scenery** | **Neutral** | **Sadness** | **Compassion** | **Fear** |
| --- | --- | --- | --- | --- | --- | --- | --- |
| 1 | F | 1.333 | 1.000 | 1.000 | 1.000 | 1.333 | 1.667 |
| 2 | M | 1.000 | 1.000 | 1.000 | 3.333 | 3.000 | 3.333 |
| 3 | M | 1.667 | 1.333 | 1.000 | 2.333 | 2.333 | 2.333 |
| 4 | F | 1.333 | 1.333 | 1.000 | 1.000 | 1.000 | 2.667 |
| 5 | M | 1.333 | 1.333 | 1.000 | 1.000 | 2.000 | 2.333 |
| 6 | F | 1.667 | 1.333 | 1.000 | 2.000 | 2.333 | 3.000 |
| 7 | M | 1.667 | 1.333 | 2.000 | 1.333 | 2.333 | 2.667 |
| 8 | M | 1.333 | 1.667 | 1.000 | 1.333 | 1.333 | 2.333 |
| 9 | F | 1.667 | 1.333 | 1.000 | 1.667 | 2.333 | 4.333 |
| 10 | M | 1.333 | 1.000 | 1.000 | 1.333 | 1.000 | 4.333 |
| 11 | F | 2.000 | 2.333 | 1.000 | 3.667 | 4.667 | 3.333 |
| 12 | F | 1.667 | 1.667 | 1.000 | 2.333 | 2.667 | 3.333 |
| 13 | F | 2.000 | 1.333 | 1.667 | 1.333 | 1.000 | 3.333 |
| 14 | M | 1.667 | 2.000 | 1.000 | 1.333 | 1.000 | 2.000 |
| 15 | F | 1.333 | 1.000 | 1.000 | 3.000 | 2.333 | 4.333 |
| 16 | M | 1.000 | 1.000 | 1.000 | 1.000 | 1.000 | 2.000 |
| 17 | F | 1.000 | 1.000 | 1.000 | 1.000 | 1.333 | 3.333 |
| 18 | M | 1.000 | 1.000 | 1.000 | 2.000 | 2.333 | 2.667 |
| 19 | F | 1.000 | 1.333 | 1.000 | 2.000 | 2.667 | 4.000 |
| 20 | M | 1.000 | 1.000 | 1.000 | 1.333 | 1.000 | 1.667 |
| 21 | F | 1.333 | 1.667 | 1.333 | 1.333 | 1.333 | 3.333 |
| 22 | M | 1.333 | 1.667 | 1.000 | 1.333 | 1.667 | 3.000 |
| 23 | F | 1.333 | 1.333 | 1.000 | 1.667 | 2.000 | 2.333 |
| 24 | M | 1.667 | 1.000 | 1.000 | 2.333 | 2.667 | 4.000 |
| 25 | F | 1.000 | 1.333 | 1.000 | 2.000 | 1.667 | 3.667 |
| 26 | M | 1.333 | 1.000 | 1.000 | 1.000 | 1.000 | 1.667 |
| 27 | F | 1.667 | 2.333 | 1.000 | 1.000 | 1.000 | 2.667 |
| 28 | M | 1.000 | 2.000 | 1.000 | 1.000 | 1.000 | 1.333 |
| 29 | F | 1.333 | 1.000 | 1.000 | 2.333 | 1.667 | 2.667 |
| 30 | M | 1.333 | 2.000 | 1.000 | 1.000 | 1.667 | 3.333 |
| 31 | F | 1.000 | 1.000 | 1.000 | 1.333 | 1.333 | 3.000 |
| 32 | M | 1.333 | 1.000 | 1.000 | 1.333 | 1.000 | 2.333 |
| 33 | F | 1.000 | 1.000 | 1.000 | 1.000 | 1.000 | 1.667 |
| 34 | F | 1.000 | 1.000 | 1.333 | 2.667 | 2.667 | 1.333 |
| 35 | F | 1.000 | 1.000 | 1.000 | 1.333 | 1.000 | 2.333 |
| 36 | F | 1.333 | 1.000 | 1.000 | 1.667 | 2.000 | 2.333 |
| 37 | F | 2.667 | 1.667 | 1.000 | 2.667 | 2.667 | 4.000 |
| 38 | F | 1.667 | 1.000 | 1.000 | 1.333 | 1.333 | 4.000 |
| 39 | M | 1.333 | 1.000 | 1.000 | 1.333 | 1.000 | 2.333 |
| 40 | M | 1.333 | 1.333 | 1.000 | 1.667 | 2.333 | 2.667 |
| 41 | M | 2.000 | 1.000 | 1.000 | 1.000 | 1.000 | 1.667 |
| 42 | M | 1.000 | 1.000 | 1.000 | 1.000 | 1.000 | 2.000 |
| 43 | M | 1.333 | 1.000 | 1.000 | 1.667 | 2.333 | 3.667 |
| 44 | M | 1.000 | 1.000 | 1.000 | 1.000 | 1.000 | 1.667 |
| 45 | F | 1.333 | 1.000 | 1.000 | 2.000 | 2.000 | 3.333 |
| 46 | F | 1.333 | 1.333 | 1.000 | 2.000 | 2.000 | 4.667 |
| 47 | F | 1.667 | 1.333 | 1.667 | 1.667 | 2.000 | 3.000 |
| 48 | F | 1.000 | 1.333 | 1.000 | 1.333 | 1.000 | 2.667 |
| 49 | F | 1.000 | 1.000 | 1.000 | 2.667 | 2.667 | 3.667 |
| 50 | F | 1.667 | 1.333 | 1.000 | 1.333 | 1.667 | 4.000 |
| 51 | F | 1.667 | 3.667 | 1.333 | 2.000 | 2.000 | 4.667 |
| 52 | F | 1.000 | 1.000 | 1.000 | 1.667 | 2.333 | 4.333 |
| 53 | F | 1.000 | 1.333 | 1.000 | 3.333 | 3.000 | 4.000 |
| 54 | F | 2.000 | 1.000 | 1.000 | 1.333 | 2.333 | 2.667 |
| 55 | F | 1.000 | 1.000 | 1.000 | 1.667 | 2.333 | 3.000 |
| 56 | F | 1.333 | 1.333 | 1.000 | 2.333 | 1.667 | 3.667 |
| 57 | F | 1.000 | 1.000 | 1.000 | 1.333 | 1.333 | 3.000 |
| 58 | F | 1.333 | 1.000 | 1.000 | 2.000 | 2.333 | 3.667 |
| 59 | F | 1.000 | 1.333 | 1.000 | 1.333 | 1.333 | 2.333 |
| 60 | F | 2.333 | 2.333 | 1.000 | 2.667 | 2.667 | 4.333 |
| 61 | F | 1.000 | 1.333 | 1.000 | 1.667 | 2.667 | 4.000 |
| 62 | F | 1.000 | 1.000 | 1.000 | 2.000 | 1.000 | 3.667 |
| 63 | F | 1.000 | 1.000 | 1.000 | 1.000 | 1.000 | 2.667 |
| 64 | M | 1.667 | 1.667 | 1.000 | 1.667 | 2.000 | 2.667 |
| 65 | M | 1.667 | 1.000 | 1.667 | 1.000 | 1.333 | 3.000 |
| 66 | M | 1.333 | 1.000 | 1.000 | 2.000 | 1.667 | 2.000 |
| 67 | M | 1.000 | 1.000 | 1.000 | 1.000 | 1.000 | 1.333 |
| 68 | M | 1.000 | 1.333 | 1.000 | 2.667 | 2.667 | 3.333 |
| 69 | M | 1.000 | 1.000 | 1.000 | 1.667 | 1.667 | 4.000 |
| 70 | M | 1.333 | 1.000 | 1.000 | 1.000 | 1.667 | 3.000 |
| 71 | M | 1.000 | 1.000 | 1.000 | 1.667 | 1.667 | 2.667 |
| 72 | M | 1.667 | 2.000 | 1.000 | 3.333 | 3.667 | 4.667 |
| 73 | M | 1.667 | 1.000 | 1.000 | 1.667 | 2.667 | 1.667 |
| 74 | M | 1.667 | 1.000 | 1.000 | 1.333 | 2.667 | 1.667 |
| 75 | M | 1.000 | 1.000 | 1.000 | 1.333 | 1.000 | 2.000 |
| 76 | M | 1.333 | 1.000 | 1.000 | 1.000 | 1.000 | 3.000 |
| 77 | M | 1.000 | 1.000 | 1.000 | 1.333 | 2.333 | 3.000 |
| 78 | M | 1.000 | 1.000 | 1.000 | 1.667 | 1.333 | 2.000 |
| 79 | M | 1.000 | 1.000 | 1.000 | 1.333 | 1.667 | 2.667 |
| 80 | M | 1.000 | 1.000 | 1.000 | 1.000 | 1.000 | 1.667 |
| 81 | M | 2.333 | 2.667 | 1.000 | 1.667 | 2.333 | 3.333 |

**S2 Table G**: Raw data of “Anxious” ratings used for statistics and figures within the manuscript

| **Participant** | **Sex** | **Erotic** | **Scenery** | **Neutral** | **Sadness** | **Compassion** | **Fear** |
| --- | --- | --- | --- | --- | --- | --- | --- |
| 1 | F | 1.000 | 1.000 | 1.000 | 1.000 | 1.000 | 1.667 |
| 2 | M | 1.333 | 1.000 | 1.333 | 3.333 | 3.333 | 3.333 |
| 3 | M | 1.667 | 1.000 | 1.333 | 2.000 | 1.333 | 2.333 |
| 4 | F | 1.667 | 1.333 | 1.000 | 1.333 | 1.000 | 2.667 |
| 5 | M | 1.000 | 1.000 | 1.000 | 1.667 | 1.667 | 2.333 |
| 6 | F | 1.667 | 1.333 | 1.000 | 2.000 | 2.333 | 3.000 |
| 7 | M | 1.333 | 1.333 | 2.000 | 1.667 | 2.000 | 3.333 |
| 8 | M | 1.000 | 1.333 | 1.000 | 1.333 | 1.000 | 2.667 |
| 9 | F | 1.667 | 1.667 | 1.000 | 2.667 | 1.667 | 4.667 |
| 10 | M | 1.333 | 1.000 | 1.000 | 2.000 | 1.333 | 5.000 |
| 11 | F | 2.000 | 2.333 | 1.000 | 3.667 | 3.333 | 3.000 |
| 12 | F | 1.333 | 1.000 | 1.000 | 1.667 | 2.333 | 2.333 |
| 13 | F | 1.333 | 1.000 | 1.000 | 1.667 | 1.333 | 3.333 |
| 14 | M | 1.000 | 1.000 | 1.000 | 1.333 | 1.000 | 2.000 |
| 15 | F | 1.667 | 1.333 | 1.000 | 2.667 | 2.333 | 3.667 |
| 16 | M | 1.000 | 1.000 | 1.000 | 1.333 | 1.333 | 2.000 |
| 17 | F | 1.000 | 1.000 | 1.000 | 1.000 | 1.000 | 2.667 |
| 18 | M | 1.000 | 1.000 | 1.000 | 2.000 | 2.000 | 2.667 |
| 19 | F | 1.000 | 1.000 | 1.000 | 1.667 | 2.333 | 3.667 |
| 20 | M | 1.000 | 1.000 | 1.000 | 1.667 | 1.000 | 1.333 |
| 21 | F | 1.000 | 1.333 | 1.000 | 1.333 | 1.667 | 3.333 |
| 22 | M | 1.333 | 2.333 | 1.333 | 2.000 | 1.667 | 3.333 |
| 23 | F | 1.000 | 1.000 | 1.000 | 1.333 | 2.000 | 3.000 |
| 24 | M | 2.000 | 1.000 | 1.000 | 1.667 | 2.000 | 3.333 |
| 25 | F | 1.000 | 1.333 | 1.000 | 1.667 | 2.667 | 4.333 |
| 26 | M | 1.000 | 1.000 | 1.000 | 1.000 | 1.000 | 1.000 |
| 27 | F | 1.000 | 1.000 | 1.000 | 2.667 | 2.000 | 3.333 |
| 28 | M | 1.000 | 1.667 | 1.000 | 1.000 | 1.000 | 1.333 |
| 29 | F | 1.667 | 1.667 | 1.000 | 2.000 | 3.333 | 2.333 |
| 30 | M | 1.000 | 1.667 | 1.000 | 1.000 | 1.333 | 3.000 |
| 31 | F | 1.333 | 1.000 | 1.000 | 1.667 | 2.000 | 2.000 |
| 32 | M | 1.333 | 1.000 | 1.000 | 1.000 | 1.333 | 1.333 |
| 33 | F | 1.000 | 1.000 | 1.000 | 1.333 | 1.000 | 2.000 |
| 34 | F | 1.000 | 1.000 | 1.000 | 2.333 | 1.667 | 1.667 |
| 35 | F | 1.000 | 1.000 | 1.000 | 2.000 | 1.667 | 2.333 |
| 36 | F | 1.667 | 1.000 | 1.333 | 3.000 | 2.000 | 3.667 |
| 37 | F | 2.000 | 1.333 | 1.000 | 3.333 | 2.333 | 4.000 |
| 38 | F | 2.000 | 1.000 | 1.000 | 1.667 | 1.000 | 4.333 |
| 39 | M | 1.333 | 1.000 | 1.000 | 1.333 | 1.000 | 1.333 |
| 40 | M | 1.000 | 1.333 | 1.000 | 1.667 | 1.667 | 1.667 |
| 41 | M | 1.333 | 1.000 | 1.000 | 1.000 | 1.333 | 1.667 |
| 42 | M | 1.000 | 1.000 | 1.000 | 1.000 | 1.000 | 2.000 |
| 43 | M | 1.000 | 1.000 | 1.333 | 2.000 | 2.333 | 3.333 |
| 44 | M | 1.000 | 1.000 | 1.000 | 1.000 | 1.000 | 2.667 |
| 45 | F | 1.000 | 1.000 | 1.000 | 2.000 | 1.667 | 3.667 |
| 46 | F | 1.000 | 1.000 | 1.000 | 2.000 | 1.667 | 4.667 |
| 47 | F | 1.333 | 1.000 | 1.333 | 1.667 | 2.000 | 2.333 |
| 48 | F | 1.000 | 1.000 | 1.000 | 2.000 | 1.000 | 3.333 |
| 49 | F | 1.667 | 1.000 | 1.000 | 3.000 | 2.667 | 4.333 |
| 50 | F | 1.000 | 1.000 | 1.000 | 1.667 | 1.000 | 4.333 |
| 51 | F | 2.000 | 3.333 | 1.667 | 2.333 | 1.667 | 4.667 |
| 52 | F | 1.333 | 1.667 | 1.000 | 2.667 | 2.667 | 4.000 |
| 53 | F | 1.000 | 1.667 | 1.000 | 2.667 | 2.000 | 4.000 |
| 54 | F | 2.667 | 1.000 | 1.000 | 1.333 | 2.000 | 2.667 |
| 55 | F | 1.333 | 1.000 | 1.000 | 2.000 | 2.000 | 3.667 |
| 56 | F | 1.000 | 1.333 | 1.000 | 1.667 | 1.333 | 3.667 |
| 57 | F | 1.000 | 1.000 | 1.000 | 1.333 | 1.333 | 2.667 |
| 58 | F | 1.667 | 1.333 | 1.000 | 2.000 | 1.667 | 3.333 |
| 59 | F | 1.000 | 1.333 | 1.000 | 1.333 | 1.000 | 2.333 |
| 60 | F | 2.000 | 3.000 | 1.000 | 2.667 | 2.667 | 4.333 |
| 61 | F | 1.000 | 1.000 | 1.000 | 1.667 | 1.000 | 4.000 |
| 62 | F | 1.333 | 1.000 | 1.000 | 2.333 | 1.667 | 4.333 |
| 63 | F | 1.000 | 1.000 | 1.000 | 1.000 | 1.000 | 3.000 |
| 64 | M | 1.333 | 2.000 | 1.333 | 2.667 | 2.667 | 3.000 |
| 65 | M | 2.333 | 1.667 | 1.000 | 1.667 | 1.667 | 2.333 |
| 66 | M | 1.000 | 1.000 | 1.000 | 2.000 | 2.333 | 2.333 |
| 67 | M | 1.000 | 1.000 | 1.000 | 1.667 | 1.333 | 2.000 |
| 68 | M | 1.000 | 1.000 | 1.000 | 3.000 | 3.000 | 3.333 |
| 69 | M | 1.333 | 1.333 | 1.000 | 1.667 | 1.333 | 3.667 |
| 70 | M | 1.333 | 1.000 | 1.000 | 1.333 | 1.667 | 2.667 |
| 71 | M | 1.000 | 1.333 | 1.000 | 2.333 | 1.667 | 3.000 |
| 72 | M | 1.667 | 2.000 | 1.333 | 4.000 | 4.000 | 4.333 |
| 73 | M | 1.000 | 1.000 | 1.000 | 1.667 | 1.667 | 2.333 |
| 74 | M | 1.000 | 1.000 | 1.000 | 1.333 | 1.000 | 1.667 |
| 75 | M | 1.000 | 1.000 | 1.000 | 1.667 | 1.333 | 2.333 |
| 76 | M | 1.667 | 1.000 | 1.000 | 1.667 | 1.667 | 3.667 |
| 77 | M | 1.000 | 1.000 | 1.000 | 2.000 | 2.667 | 4.000 |
| 78 | M | 1.000 | 1.000 | 1.000 | 2.000 | 2.000 | 2.000 |
| 79 | M | 1.000 | 1.000 | 1.000 | 1.333 | 2.667 | 3.000 |
| 80 | M | 1.333 | 1.000 | 1.000 | 1.000 | 1.000 | 1.333 |
| 81 | M | 1.000 | 2.000 | 1.000 | 3.333 | 4.000 | 4.667 |
